# Graph Convolutional Network Using Adaptive Neighborhood Laplacian Matrix for Hyperspectral Images with Application to Rice Seed Image Classification

**DOI:** 10.3390/s23073515

**Published:** 2023-03-27

**Authors:** Jairo Orozco, Vidya Manian, Estefania Alfaro, Harkamal Walia, Balpreet K. Dhatt

**Affiliations:** 1 University of Puerto Rico at Mayaguez, Mayagüez, PR 00681, USA; 2 University of Nebraska-Lincoln, Lincoln, NE 68583, USA

**Keywords:** graph convolutional network, adaptive neighborhood, laplacian matrix, hyperspectral image classification, hyperspectral rice seed images

## Abstract

Graph convolutional neural network architectures combine feature extraction and convolutional layers for hyperspectral image classification. An adaptive neighborhood aggregation method based on statistical variance integrating the spatial information along with the spectral signature of the pixels is proposed for improving graph convolutional network classification of hyperspectral images. The spatial-spectral information is integrated into the adjacency matrix and processed by a single-layer graph convolutional network. The algorithm employs an adaptive neighborhood selection criteria conditioned by the class it belongs to. Compared to fixed window-based feature extraction, this method proves effective in capturing the spectral and spatial features with variable pixel neighborhood sizes. The experimental results from the Indian Pines, Houston University, and Botswana Hyperion hyperspectral image datasets show that the proposed AN-GCN can significantly improve classification accuracy. For example, the overall accuracy for Houston University data increases from 81.71% (MiniGCN) to 97.88% (AN-GCN). Furthermore, the AN-GCN can classify hyperspectral images of rice seeds exposed to high day and night temperatures, proving its efficacy in discriminating the seeds under increased ambient temperature treatments.

## 1. Introduction

Hyperspectral images (HSI) contain rich spectral and spatial information useful for material identification. Recently, many methods based on deep learning tools have been developed resulting in an increase in the classification accuracy and segmentation precision of these images [1]. One of these methods is the use of graphs together with deep learning. Many data structures that are non-Euclidean can be represented in the form of graphs [2,3]. Nowadays sophisticated sensing and imaging technologies are available for the acquisition of complex image datasets. The resultant images have higher information content requiring non-Euclidean space representations. Therefore, the use of graph theory combined with deep learning in images has proved to be more effective for processing these images. Based on convolutional networks and deep networks, the concept of Graph Convolutional Networks (GCN) has been developed and applied to image [4]. An image with a regular domain (regular grid in the Euclidean space) is represented as a graph, where each pixel in the image represents a node, and edges are the connections between adjacent nodes [5]. GCNs are used to model long-range spatial relationships in HSI where a CNN fails [6]. One of the most important parts of the development of the GCNs is the generation of the adjacency matrix and the derivation of the Laplacian [7,8,9]. The adjacency matrix of undirected graphs represents the relationship between vertices, in this case pixels. Quin et al. [10] proposed a GCN method that markedly improved the classification accuracy in HSI by taking the spatial distance between nodes and multiplying it by the adjacency matrix which contained spectral signature information. L Mou et al. [11] built the graph by using correlation to measure the similarity among pixels and thereby identifying pixels belonging to the same category. They built a two-layer GCN that improved the classification results. A drawback of using GCN is the computational cost involved in constructing the adjacency matrix. To address this problem, the authors in [12] constructed an adjacency matrix using pixels within a patch containing rich local spatial information instead of computing the adjacency matrix using all the pixels in the image. Another important work in reducing the computational cost is the one proposed by Hong et al. in [6], where the authors built a method called MiniGCN, which allows training large-scale GCN with small batches. The adjacency matrix is constructed using K-nearest neighbors, taking the 10 nearest neighbors of the central pixel into consideration. For representing a HSI as a graph, many authors rely on building the adjacency matrix using k-nearest neighbors [13,14], because it is an easy method to understand and apply, but this method does not sufficiently capture the spectral characteristics of hyperspectral data [15]. The superpixel approach [16,17] creates the graph from superpixel regions to reduce the graph size.

Some authors have implemented neighbor selection using threshold, for example, in hyperspectral unmixing applications [18]. Others have implemented adaptive shapes [19] in classification methods that does not involve GCNs. In this work, we propose a novel way of adaptively creating the adjacency matrix based on a neighbor selection approach called AN-GCN. The general idea is to iterate over each pixel, aggregating neighbors that belong to the same class as the central pixel based on a variance measure creating an adjacency matrix. The algorithm selects a different number of neighbors for each pixel thereby adapting to the spatial variability of the class in which the pixel belongs with the aim of improving the classification and solving the uncertainty problem that arises due to border pixels. The construction of the adjacency matrix is a crucial step for a GCN to succeed in HSI classification [13]. The performance of AN-GCN is compared with GCN methods that do not combine other machine learning stages in the architecture. An optimal graph representation of the HSI as the adjacency matrix impacts the classification result significantly. Recently, hyperspectral images are being used in agriculture for the analysis of rice seeds [20,21,22], because an accurate phenotype measurement of the seeds using non-destructive methods helps in evaluating the quality of the seeds, and contributing to improvement in agricultural production [23]. Another reason for using hyperspectral images for the classification of rice seeds is to save work and time, since these processes are conventionally done manually by expert inspectors in the area [20,24]. Hyperspectral rice seed images have been classified using only conventional machine learning methods, and there is no report of using GCN based methods. To test the effectiveness of AN-GCN method in classifying hyperspectral images of rice seeds, less than 10% of the rice image data is used for training the AN-GCN and the remaining is used for testing and validation. The main contributions of this work are (1) a novel way of computing the adjacency matrix using adaptive spatial neighborhood aggregation to improve the performance of GCN in HSI classification; (2) Performing classification of rice seed HSIs grown under high temperatures using the AN-GCN approach.

## 2. Materials and Methods

This section presents the AN-GCN method based on the construction of the adjacency matrix by adaptive neighbor selection. The goal is to characterize the homogeneity between pixels for better discrimination between classes. Pixels that have a degree of homogeneity most likely belong to the same class. For this, a variation metric is used to measure homogeneity [25].

(1) $$S^{2}=\sum (X_{i}-\bar{X})/n-1,$$

where $X_{i}$ are the pixel intensity values per band that belong to a radius R, and *n* is the number of bands in each pixel.

**Figure 1:**
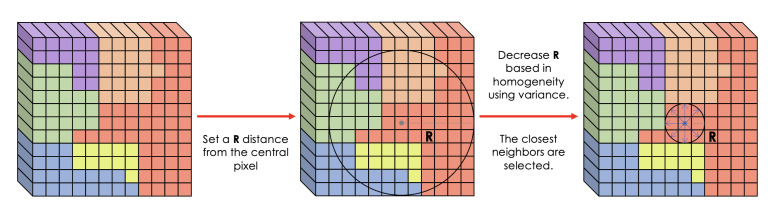
Scheme of neighbors selection using a variance radius.

A radius R is selected based on unit distances using spatial coordinates as is shown in the scheme of Figure 1. If R = 1, the algorithm selects only the four closest neighbors, a higher value of radius would select more neighbors. An initial radius is set to R = 100. In order to select homogeneous regions, a variance measure is applied to the area covered by radius R. The threshold values shown in Table 1 is applied to the variance value for the different HSIs for decreasing the area covered by radius R, so that only pixels from homogeneous neighborhoods are selected.

If the variance is larger than the threshold value, the coverage radius is decreased until the desired variance threshold is reached. If the variance is smaller than the threshold, the selected neighbors meet the criterion, and the pixels in the neighborhood belong to the same class, thereby resulting in successful class discrimination.

The threshold value for each HSI is determined by calculating the average variance of the set of pixels that are known to belong to the same class, then a unique threshold is chosen for all classes. The threshold value is different for each HSI. The neighbor selection is applied to each pixel of the HSI. Each pixel will have a different neighborhood depending on the homogeneity of the region surrounding it. Once the neighborhood region with the pixel neighbors for each center pixel is selected, the adjacency matrix $A_{ad}$ is built using the radial basis function (RBF).

(2) $$A_{i,j}=Exp(∥x_{i}-x_{j}∥)/\sigma^{2},$$

where $x_{i}$ and $x_{j}$ are the pixel intensity vectors per band for pixels *i* and *j*, $\sigma^{2}$ is a control parameter.

After the construction of the adjacency matrix, the GCN algorithm is applied. The Laplacian matrix computed from the adaptive neighborhood adjacency matrix is used to measure how much the function value is changing at each node (graph gradient).

(3) $$L_{ad}=I_{n}-D^{-\frac{1}{2}}A_{ad}D^{-\frac{1}{2}},$$

where *D* is a diagonal matrix of node degrees and *A* is the adjacency matrix. The normalized Laplacian matrix is positive semi-definite, and with this property the Laplacian can be diagonalized by the Fourier basis *U*, therefore the Equation (3) can be written as $L_{ad}=U$Λ$U^{T}$, where *U* is a matrix of Eigenvectors and Λ is a diagonal matrix of Eigenvalues λ. The Eigenvectors satisfy the orthonormalization property $UU^{T}=I$. The graph Fourier transform of a graph signal *X* is defined as $F\left(X\right)=U^{T}X$ and the inverse $F{\left(X\right)}^{-1}=U^{T}\hat{X}$, where *X* is a feature vector of all nodes of a graph. Graph Fourier transform makes a projection of the input graph signal to an orthonormal space whose bases is determined from the Eigenvectors of the normalized graph Laplacian [5]. In signal processing the graph convolution process with a filter *g* is defined as:

(4) $$X*g=F{\left(X\right)}^{-1}\left(F\left(X\right)⊙F\left(g\right)\right),$$

where ⊙ is the element-wise product. The filter is defined by $g=diag(U^{T}g)$ and the convolution Equation (4) using the transform of a graph signal is simplified as

(5) $$X*g=UgU^{T}X,$$

due to the computational complexity of Eigenvector decomposition in Equation (5), and as the filter *g* is not localized, which means that it may take nodes far away from the central node, Hammond et al. [26], approximate *g* using *K*th order truncated expansion of Chebyshev polynomials. The Chebnet Kernel is defined as

(6) $$g=\sum_{i=0}^{k}\theta_{i}T_{i}\left(\hat{\Lambda }\right),$$

where *i* represents the smallest order neighborhood, $\theta_{i}$ are the Chebyshev coefficients, *k* represents the largest order neighborhood, *T* is the Chebyshev polynomial of the *k*th order and $\hat{\Lambda }=2\Lambda /\lambda_{max}-I$. Replacing the filter of Equation (6) in the convolutional Equation (5) is obtained,

(7) $$X*g=U\left(\sum_{i=0}^{k}\theta_{i}T_{i}\left(\hat{\Lambda }\right)\right)U^{T}X,$$

(8) $$X*g=\sum_{i=0}^{k}\theta_{i}UT_{i}\left(\hat{\Lambda }\right)U^{T}X,$$

As $UT_{i}\left(\hat{\Lambda }\right)U^{T}=T_{i}\left(\hat{L}\right)$, the Equation (8) can be written as

(9) $$X*g=\sum_{i=0}^{k}\theta_{i}T_{i}\left(\hat{L}\right)X,$$

where $\hat{L}=2L/\lambda_{max}-I$ and taking k=1 and the largest eigenvalue of $\lambda_{max}=2$ [27], Equation (9) can be written as:

(10) $$X*g=(\theta_{0}T_{0}\left(\hat{L}\right)+\theta_{1}T_{1}\left(\hat{L}\right))X,$$

where $T_{0}\left(\hat{L}\right)=1$ and $T_{1}\left(\hat{L}\right)=L$, the Equation (10) becomes:

(11) $$X*g=(\theta_{0}+\theta_{1}L)X,$$

To avoid over-fitting GCN assumes $\theta =\theta_{0}=\theta_{1}$, and replacing the Equation (3) for *L* in Equation (11), the following equation is obtained:

(12) $$X*g=\theta (I+D^{-\frac{1}{2}}A_{ad}D^{-\frac{1}{2}})X,$$

Therefore, using a normalization proposed by [27] $I+D^{-\frac{1}{2}}A_{ad}D^{-\frac{1}{2}}\to {\hat{D}}^{-\frac{1}{2}}\hat{A_{ad}}{\hat{D}}^{-\frac{1}{2}}$ where $\hat{A_{ad}}=A_{ad}+I$ and ${\hat{D}}_{ii}=\sum_{j}{\hat{A_{ad}}}_{ij}$. GCN uses a propagation rule for updating the weights in the hidden layer iteratively until the output of the GCN converges to the target classes. The propagation rule for GCNs is:

(13) $${\hat{D}}^{-\frac{1}{2}}\hat{A_{ad}}{\hat{D}}^{-\frac{1}{2}}X\Theta ,$$

where Θ is a matrix of filter parameters (weigths). Most articles related to GCN represent the propagation rule as follows:

(14) $$H_{l+1}=\sigma \left({\hat{D}}^{-\frac{1}{2}}\hat{A_{ad}}{\hat{D}}^{-\frac{1}{2}}H^{\left(l\right)}W^{\left(l\right)}+b^{\left(l\right)}\right),$$

where matrix $H^{\left(l\right)}$ denotes the features output in the *l*th layer (input). σ is the activation function, $W^{\left(l\right)}$and $b^{\left(l\right)}$ are the learned weights and biases.

In order to reduce the computational cost of AN-GCN, batch processing of MiniGCN [6] is followed. The batches of pixels are extracted as in CNNs, followed by the construction of subgraphs for each batch from the adjacency matrix $A_{ad}^{b}$. The propagation rule described in Equation (14) for each subgraph is:

(15) $${{\hat{H}}^{b_{i}}}_{l+1}=\sigma \left({\hat{D^{b_{i}}}}^{-\frac{1}{2}}\hat{A_{ad}^{b_{i}}}{\hat{D^{b_{i}}}}^{-\frac{1}{2}}{H^{b_{i}}}^{\left(l\right)}{W^{b_{i}}}^{\left(l\right)}+{b^{b_{i}}}^{\left(l\right)}\right),$$

where $b_{i}$ are the batches of pixels or subgraphs used for network training. The final output of the propagation rule is a vector that joins all the subgraph outputs.

(16) $${\hat{H}}_{l+1}=\left[{H^{b1}}_{\left(l+1\right)},{H^{b2}}_{\left(l+1\right)},{H^{b3}}_{\left(l+1\right)}...{H^{bN}}_{\left(l+1\right)}\right],$$

**Figure 2:**
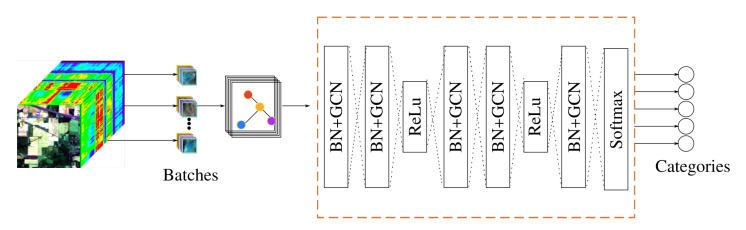
AN-GCN architecture.

The pseudocode for creation of the adjacency matrix for AN-GCN is described in Algorithm 1 and the pseudo-code for the AN-GCN is given in Algorithm 2. Figure 2 shows the architecture of AN-GCN. Batch normalization is implemented before GCN layer.
**Algorithm 1** Adjacency Matrix for AN-GCN**  1** **Input:** Original HSI, Ground Truth**  2** **for** Each pixel in HSI **do****  3**        Set a radius **R****  4**        Find Homogeneity of pixels inside **R** using variance**  5**        **while** Variance > threshold value **do****  6**              Decrease R until meet the desired threshold**  7**              **If R** < 1 **then** Set minimum possible radius to 1**  8**              **end if****  9**        **end while****10**        Calculate weights of pixel inside **R** that meet the criterion using Equation (2)**11** **end for****12** Construct Adjacency matrix**13** Compute the Laplacian matrix

**Figure 3:**
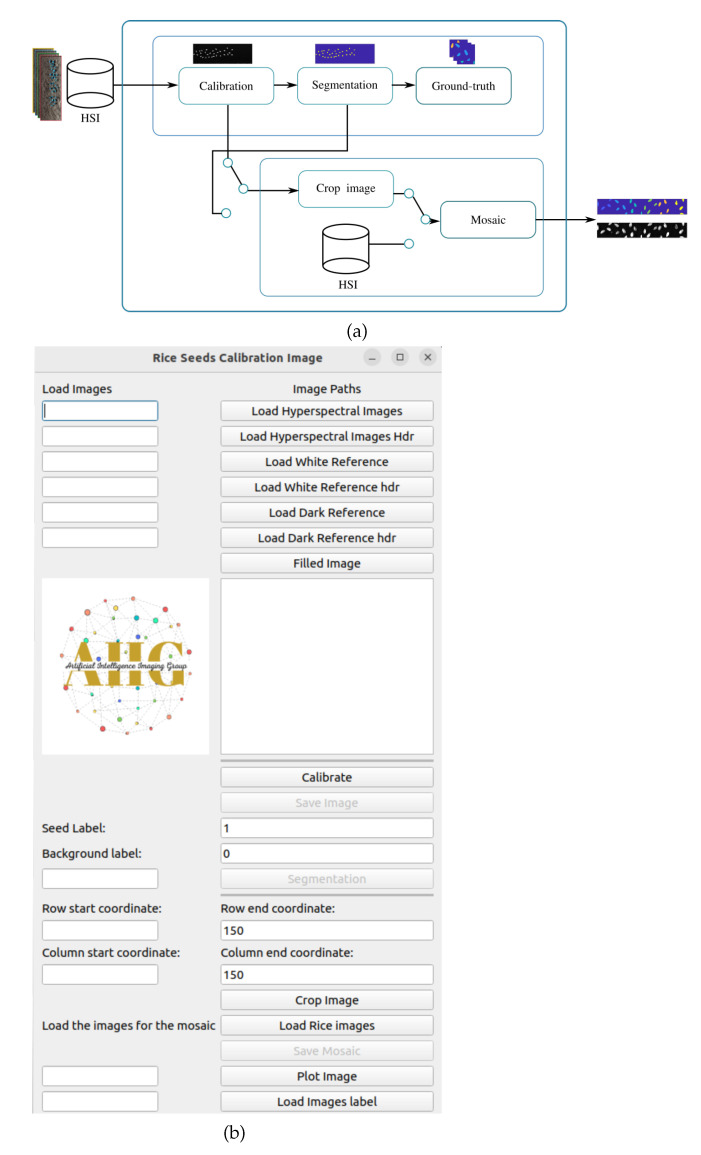
(**a**) Workflow for HSI seed images and ground truth mosaic construction, (**b**) HSI seed calibration application.

    A graphical user interface is developed for constructing the image and ground truth mosaics for the pixel-based classification of rice seed HSIs. The hyperspectral rice seed images are calibrated using the workflow illustrated in Figure 3a. The input is a rice seed hypercube 3 dimensional array.    
**Algorithm 2** Pseudo code of AN-GCN for HSI classification**  1** **Input:** Original HSI, Laplacian Matrix *L*, Labels, epochs = 200, batch size = 100**  2** Initialize **W** and **b** parameters**  3** **for** Each batch of pixels **do****  4**        ${{\hat{H}}_{b}}^{l+1}=\sigma \left({\hat{D_{b}}}^{-\frac{1}{2}}\hat{A_{b}}{\hat{D_{b}}}^{-\frac{1}{2}}{H_{b}}^{\left(l\right)}{W_{b}}^{\left(l\right)}+{b_{b}}^{\left(l\right)}\right)$**  5**        Return ${{\hat{H}}_{b}}^{l+1}$, Loss**  6**        Softmax**  7**        Optimize loss function using Adam**  8**        Update parameters**  9** **end for****10** **Output:** Predicted label for each pixel using argmax.

where$I_{c}$ represents the calibrated image, $I_{d}$ is the dark reference, and $I_{w}$ is the white reference acquired by the sensor. After calibration, the image is segmented using two methods. The first is the Otsu thresholding algorithm which is applied to identify the seeds from the background. In the second step, the segmentation is improved using a Gaussian filter with a σ=0.7, and the labeling is performed using 2 nearest neighbor connectivity. The “ground truth block” generates the labels for the seeds and the background. In addition, a “crop image block” is available to select a Region Of Interest (ROI), from an input calibrated or segmented image. Once the images are cropped, a parallel block is implemented to create the mosaics for training and classification using the GCN architecture. A Hyperspectral Seed Application has been developed to perform the above processes. The GUI of the App is illustrated in Figure 3b, and is implemented in python using pyqt5 libraries.

The GUI is operated in the following manner. The user uploads the HSI of the seed and the white and dark references in the provided widgets. There are buttons for calibrating and saving the image. The user has the option to assign categorical labels to the seed classes, which can be based on the temperatures the seeds are exposed to, or different varieties of seeds. Once the integer labels are selected, the images are labeled and saved. Another useful function provided by the GUI is for cropping an HSI. As HSIs are large and occupy more space, the user can crop the images by specifying the row and column coordinates enclosing the seed. Finally, a mosaic with seed images of different categories is created by concatenating the individual images in the horizontal or vertical direction. A button is provided for visualization of the images at any stage.

### Datasets

Four hyperspectral image datasets are used to test the proposed AN-GCN method. The training set is generated by randomly taking pixels in each class, the class 0 corresponding to the backgroung is not considered for training. Once the pixels for training and testing are selected, the adjacency matrix corresponding to the training and testing pixels is also selected. Table 2 gives the specification for each dataset.

**Figure 4:**
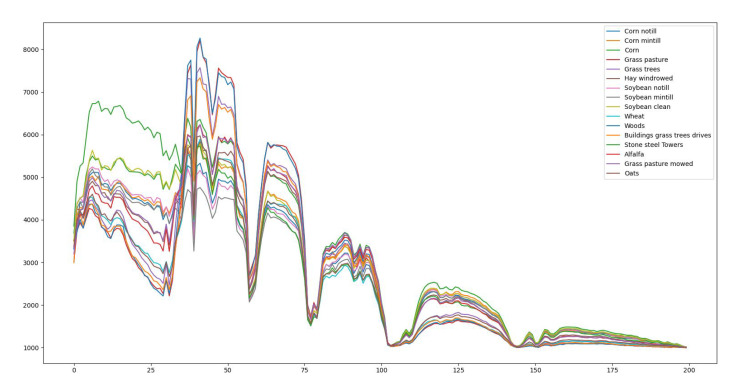
Spectrum of each material in the Indian Pines hyperspectral image.

(1) *Indian Pine Dataset*: This scene is taken from North-Western Indiana with an Airborne Visible/Infrared Imaging Spectrometer (AVIRIS) optical sensor over the Indian pine test area. Each band contains 145 × 145 pixels with a total of 224 bands. Indian Pine scene contains 16 classes. The spectrum for a pixel from each of the 16 classes is shown in Figure 4. Among the classes are crops, vegetation, smaller roads, highways, and low-density housing, which are named in Table 3 along with the training and testing set used for training and testing the AN-GCN. 20 noisy bands due to water absorption are removed leaving a total of 200 bands.

(2) *University of Houston dataset*: This hyperspectral dataset is acquired using an ITRES-CASI sensor. It contains 144 spectral bands between 380–1050 nm; with a spatial domain of 349 × 1905 pixels per band and a spatial resolution of 2.5 m. The data is taken from the University of Houston campus and contains the land cover and urban regions with a total of 15 classes as shown in Table 4, along with the training and testing set used for training and testing the AN-GCN.

(3) *Botswana dataset*: This scene is taken over the Okavango Delta, Botswana in 2001–2004 using an EO-1 sensor. It contains 242 bands between 400–2500 nm. After the denoising process is applied, 145 bands remain [10–55, 82–97, 102–119, 134–164, 187–220]. Each band contains 1476 × 256 pixels with a spatial resolution of 2.5 m.

(4) *Rice Seeds dataset*: Hyperspectral images of rice seeds grown under high day/night temperature environments, and control environment are taken with a high-performance line-scan image spectrograph (Micro-Hyperspec${}^{®}$Imaging Sensors, Extended VNIR version) [23]. This sensor covers the spectral range from 600 to 1700 nm. The dataset contains 268 bands and 150 × 900 pixels per band. there are four temperature treatments shown in Table 5.

**Figure 5:**
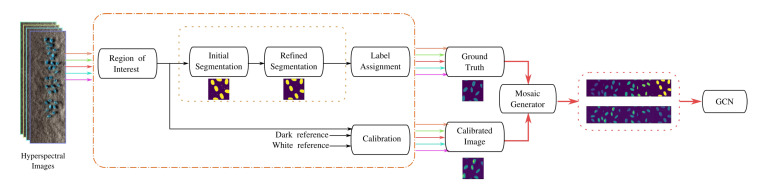
Workflow for preprocessing of rice seed hyperspectral images.

The rice seed images are calibrated using the workflow outlined in Figure 5. The workflow is composed of five stages described as follows: the first stage consists of reading the input image and selecting a region of interest (ROI) containing rice samples. Once the ROI is obtained, an initial segmentation based on histogram selection is performed. The rice seeds are thresholded from the background, if their intensities $x_{h}$ are within the interval $50<x_{h}\geq 100$.

The initial segmentation extracts the rice seeds in the regions of interest. However, some isolated pixels belonging to the background class are present within the rice seed region. To remove these pixels a Gaussian filter with a standard deviation of 0.7 is applied to the cropped image. In addition, similar regions are connected using connected component labeling using k-connectivity. Here, a 3 × 3 kernel with 2-connectivity is used to connect disconnected regions and assign a label. Once the refinement of the segmentation is performed, the label assignment stage assigns labels to each pixel using the tuple (0, class number), where 0 represents the background.

For rice seed HSI, two types of classification were made. The first is to classify each treatment by exposure time, that is, for the same time, the different treatments are taken as a class, where the class number is assigned as follows: 1 for HDT class, 2 for HDNT1 class, 3 for HDNT2 class, 4 for HNT class, and finally 5 for Control class. The second type of classification was to classify the rice seeds for different exposure times for the same treatment; for this case, the classes were assigned as follows: 1 for 168 class, 2 for 180 class, 3 for 204 class, 4 for 216 class, 5 for 228 class and finally 6 for 240 class.

The images are calibrated using the Shafer Model [28] described in Equation (17), $Ic_{\lambda }$ is the constant reflection value at a predetermined wavelength, $I_{\lambda }$ is the measurement of reflection, $W_{l}$ is the white reference obtained from the calibration of a Teflon tile, and B is the black reference.

(17) $$Ic_{\lambda }=\frac{I_{\lambda }-B}{W-B}$$

The rice seed HSI preprocessing workflow is performed for the five classes of images. The last stage is the mosaic generator which stitches the five groundtruth images, and the five calibrated HSIs into two mosaics, respectively. These two mosaics are then input to the GCN for classification.

## 3. Results

The performance of the AN-GCN method is evaluated by comparing its classification results with pure GCN based results reported by other authors in the literature, especially MiniGCN. Three metrics: Overall Accuracy (OA), Average Accuracy (AA), and Kappa score are used for this comparison. Increased accuracies using AN-GCN are highlighted in bold in the Tables. To further test the effectiveness of AN-GCN, several tests are done using fixed nearest neighbors and compared with the proposed method of adaptive neighbors.

**Figure 6:**
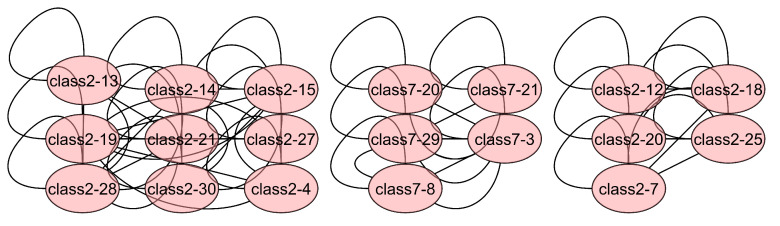
Subgraphs of Laplacian matrix from Botswana dataset.

To demonstrate that the adaptive neighbor selection method aggregates pixels that belong to the same class, the Laplacian matrix used for training the Botswana scene is plotted. This scene is taken since it is a small matrix compared to the other HSI images and the subgraphs belonging to the classes can be plotted. In Figure 6, a portion of the matrix is shown, where the subgraphs (pixels) belonging to the same class can be visualized. For example, pixels 20, 21, 29, 3 and 8 belong to class 7, corresponding to Hippo grass class.

A test is carried out to show that the construction of the adjacency matrix influences the final classification. For this test, the Indian Pine scene is used as a reference and the adjacency matrix is built using fixed neighbors, starting by taking the first 4 neighbors for each pixel. Then the matrix is built for 8 neighbors, 12, 20 neighbors and as a final step the matrix is built with the proposed method as shown in Table 6. Once the different matrices are built, each one is used in the GCN classification model. In the GCN training process, the same parameters shown in Algorithm 2 are used for each of the matrices and the final classification results of Overall Accuracy (OA), Average accuracy (AA) and kappa score using different numbers of neighbors are shown in Table 6.

**Figure 7:**
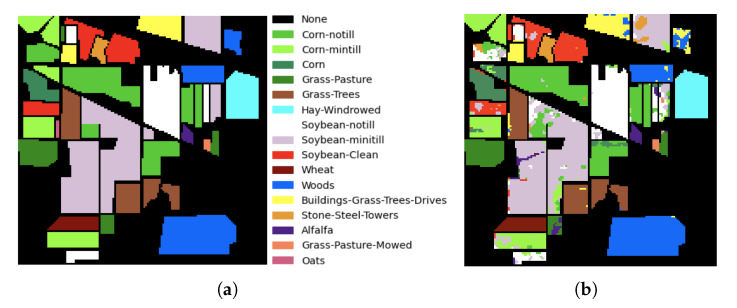
Classification map in Indian Pine dataset. (**a**) Groundtruth map. (**b**) AN-GCN.

For the construction of the adjacency matrix for the Indian Pine scene, a variance threshold of 0.16 is used for the selection of neighbors. The classification results are shown in Table 7, obtaining a perfect classification for classes: wheat, stone-steel-towers, alfalfa, grass-pasture-mowed and oats. Figure 7 shows the Indian Pines classification map where the different pixel classification results can be seen.

The results obtained for Houston university are reported in Table 8. The AN-GCN obtains the best classification values of OA, AA, Kappa score compared to the other reported GCN methods. A perfect classification is obtained for classes: healthy grass, synthetic grass, soil, water, tennis court and running track.

**Figure 8:**
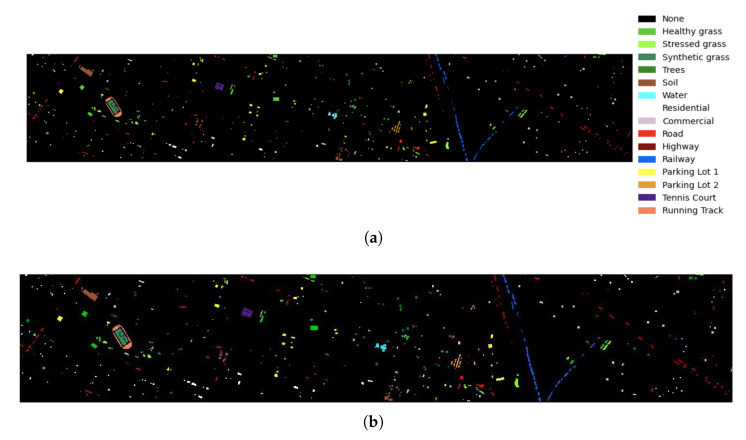
Classification map for Houston university. (**a**) Groundtruth map. (**b**) AN-GCN.

Figure 8 shows the classification map of the AN-GCN method for the Houston university scene, where Figure 8a is the groundtruth and Figure 8b is the classification map using the proposed model.

Table 9 shows the training and testing set used for the Botswana HSI and reports the classification accuracies obtained this dataset. For the construction of the adjacency matrix, a threshold of 0.024 is used, shown in the Table 1. The results show that AN-GCN obtains the highest accuracy values for 13 of the 14 classes that make up the Botswana scene. The highest reported classification accuracy of AA (99.2%), OA (99.11%) and Kappa score (0.9904) are obtained with the AN-GCN method.

For HSI rice seeds the classification results are shown in Table 10 and Table 11. The first table corresponds to the classification results by treatment for a specific time, the exposure time of 204 and 228 h had the best OA, AA and Kappa score values and time 240 had the lowest values.

Table 11 corresponds to the classification results for the different exposure times for the same treatment. The HDT treatment obtained the best OA, AA, and Kappa score classification values.

**Figure 9:**
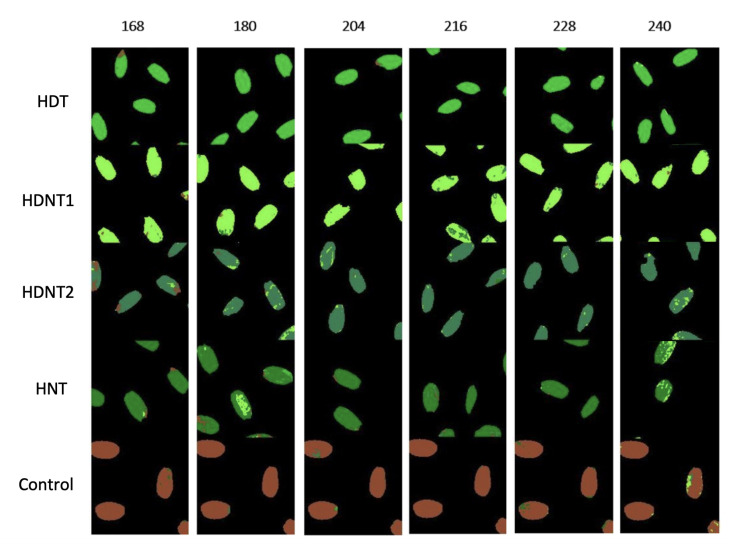
Classification map of HSIs of rice seeds from different hours of exposure for each temperature treatment.

**Figure 10:**
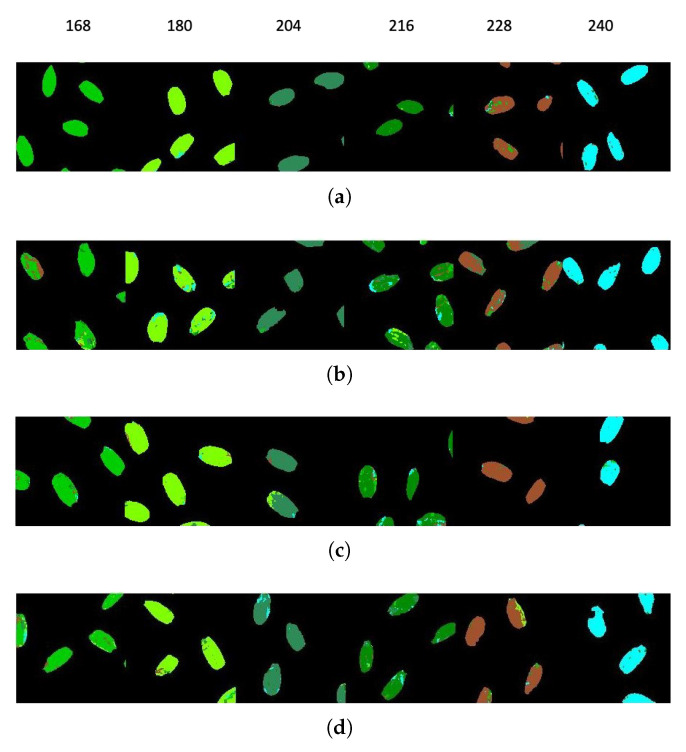
Classification map for rice HSI datasets for different temperature treatments: (**a**) HDT, (**b**) HDNT1, (**c**) HNT, (**d**) HDNT2.

Figure 9 shows the classification map for the different classes at a specific time and Figure 10 shows the classification map for the different exposure times for the same treatment.

## 4. Discussion

### 4.1. Indian Pines

The results obtained for the classification of the Indian Pines scene are reported in Table 7. A considerable improvement in classification accuracy using AN-GCN can be seen when comparing with other GCN methods, especially the reported MiniGCN methods [6,29,30]. AN-GCN method improves the accuracy by more than 13 percent affirming the importance of adjacency matrix creation. Incorporating neighborhood information adaptively results in better discrimination between classes, especially in the boundary region between classes. The classification map is shown in Figure 7. For a better visual comparison, the Indian Pine groundtruth is provided in Figure 7a and the classification map from the AN-GCN method is shown in Figure 7b. Compared to the groundtruth, it can be seen that some pixels of the Wood class (blue color) are misclassified into Building-grass-trees drives (yellow color) because these classes have similar spectral signatures. However, the AN-GCN classification map looks similar to the groundtruth map with minimal misclassification errors.

Table 6 gives the results obtained for the test samples from the Indian Pines scene, showing that the adaptive spatial neighbor selection performs better than fixed neighborhood selection. Another test to check that there is better class discrimination using adaptive neighbors is to plot the training Laplacian matrix. In Figure 6, a subgraph of the Laplacian matrix for the Bostwana dataset is shown. It is observed that there are subgraphs grouping pixels that belong to the same class showing the effectiveness of the adaptive neighbors in aggregating pixels of the same class and avoiding pixels of a different class in being grouped together thereby improving the training of the GCN and the final classification results of the HSI.

### 4.2. Houston University

The training and testing set and the classification accuracies obtained for the Houston university dataset are reported in Table 8. The results show an increase in classification accuracy with respect to the other GCN-based methods. The minimum classification accuracy for the AN-GCN method is 90.88% corresponding to the parking lot 2 class, showing the effectiveness of this method compared to the other methods. One of the classes that has the lowest classification accuracy using almost all methods is the Commercial class. This behavior is also reported in [16]. However, with the AN-GCN method, this class achieves a higher classification accuracy of 97.06%, showing that AN-GCN has superior performance compared to the other methods. In Figure 8a,b, the groundtruth and the classification map obtained using the AN-GCN method are shown, respectively. Due to the improvement of classification performance using the AN-GCN method, it is difficult to observe the misclassification of some pixels. However, when observing the right side of the classification map in detail, some missed pixels can be identified.

### 4.3. Botswana

The classification results for the Botswana Scene are given in Table 9, along with the number of training pixels and testing pixels used. Botswana presents outstanding results using the AN-GCN method having a higher value of overall accuracy, average accuracy, and Kappa score compared to the other methods. There are only a few reported classification results using GCN for the Botswana dataset, however, the AN-GCN method is efficient in classifying this dataset.

### 4.4. Rice Seeds

The GCN architecture performs well in classifying the rice seed HSIs from different temperature treatments, as well as classifying the seeds from subclasses of six temperature exposure duration. The GCN integrates spatial and spectral information adaptively performing a pixel-based classification obtaining a good classification performance for all four temperature treatments as well as for sub-classes of varying temperature exposure duration.

The classification results shown in Table 11, show lower values in the classification than those shown in Table 10. These results are expected, since for this case the different exposure hours are being taken from the same treatment, but even so, the proposed method is able to discriminate the exposure times for the same treatment, showing that the rice seed undergoes changes as the exposure time to high temperature increases. The rice seed HSIs from the highest day and night temperature of 36/32 degree Celsius give the lowest accuracy, showing that higher temperatures alter the seed spectral-spatial characteristics drastically making it indiscriminable from other treatment classes.

Comparing these results with those reported in [23], the authors had obtained a classification accuracy of 97.5% only for two classes using a 3D CNN with 80% of the data for training. While using the AN-GCN method, four treatments classes and six temperature exposure duration classes are classified using only 10% of the data for training giving satisfactory classification results.

## 5. Conclusions

This paper presents a new method for neighborhood aggregation based on statistics which improves the graph representation of high spectral dimensional datasets such as hyperspectral images. This method performs better than recent state-of-the-art implementations of GCNs for hyperspectral image classification. The presented method selectively adapts to the spatial variability of the neighborhood of each pixel and hence acts as a significant measure of discriminability in localized regions. An increased classification accuracy of 4% is obtained with the University of Houston and Botswana datasets, and an increase in accuracy of 1% is obtained with the Indian Pine dataset. The AN-GCN method successfully characterizes intrinsic spatial-spectral properties of rice seeds grown under higher than normal temperatures and classifies hyperspectral images of these seeds with high precision using less than 10% of the data for training, placing the AN-GCN as a preferable method for agricultural applications compared to other CNN-based methods.

## Figures and Tables

**Table 1:** Threshold values for selecting neighborhood pixels for each HSI.

Scenes	Threshold Value
Indian Pine	0.16
University of Houston	0.25
Botswana	0.026
Rice seeds	0.16

**Table 2:** Hyperspectral dataset specifications.

Scene	Spatial Size (Pixels)	Spatial Resolution	Spectral Size (Bands)	Spectral Resolution (nm)	Sensor
**Indian Pines**	145 × 145	20 m pixels	200	400–2500	AVIRIS
**Houston University**	349 × 1905	2.5 m pixels	144	380–1050	ITRES-CASI
**Botswana**	1476 × 256	30 m pixels	145	400–2500	HYPERION EO-1
**Rice seed**	150 × 900	1100–1600 pixels	268	600–1700	Micro-Hyperspec${}^{®}$Imaging

**Table 3:** Number of training and testing samples for the different classes in Indian Pine dataset.

Class No.	Class Name	Training	Testing
1	Corn Notill	50	1384
2	Corn Mintill	50	784
3	Corn	50	184
4	Grass pasture	50	447
5	Grass trees	50	697
6	Hay Windrowed	50	439
7	Soybean Notill	50	918
8	Soybean Mintill	50	2418
9	Soybean clean	50	564
10	Wheat	50	162
11	Woods	50	1244
12	Buildings Grass Trees Drives	50	330
13	Stone Steel Towers	50	45
14	Alfalfa	15	39
15	Grass Pasture Mowed	15	11
16	Oats	15	5
	total	695	9671

**Table 4:** Number of training and testing samples for the different classes in Houston University dataset.

Class No.	Class Name	Training	Testing
1	Healthy grass	198	1053
2	Stressed grass	190	1064
3	Synthetic grass	192	505
4	Tree	188	1056
5	Soil	186	1056
6	Water	182	143
7	Residential	196	1072
8	Commercial	191	1053
9	Road	193	1059
10	Highway	191	1036
11	Railway	181	1054
12	Parking lot 1	192	1041
13	Parking lot 2	184	285
14	tennis court	181	247
15	Running track	187	473
	Total	2832	12,197

**Table 5:** Temperature treatments for rice seed HSI dataset.

Treatment Class	Day/Night Temperature ${}^{\circ }$C
Control	28/23
HDNT2 (High day night temperature 2)	36/28
HDNT1 (High day night temperature 1)	36/32
HDT (High day temperature)	36/23
HNT (High night temperature)	30/28

**Table 6:** Comparison of classification using different K-nearest neighbors in Indian Pines dataset.

K-Nearest Neighbors	OA (%)	AA (%)	Kappa Score
k = 4	82.71	87.12	0.8003
k = 8	79.14	85.53	0.7568
k = 12	78.91	83.49	0.7555
k = 20	75.47	76.62	0.7121
**Adaptive** **Neighborhood**	**88.36**	**91.13**	**0.8453**

**Table 7:** Classification performance (%) of various GCN methods for Indian Pines dataset.

Class No.	MIniGCN [29]	GCN [27]	MiniGCN [30]	GCN [12]	Non-Local GCN [11]	MiniGCN [6]	AN-GCN
1	79.12 ± 7.04	56.71 ± 4.42	68.07	53.54	**89.03**	72.54	85.04
2	56.13 ± 6.46	51.50 ± 2.56	53.97	53.01	**100.00**	55.99	81.76
3	22.16 ± 16.37	84.64 ± 3.16	66.84	87.77	93.51	92.93	**95.65**
4	**91.80 ± 1.10**	83.71 ± 3.20	77.37	90.89	94.12	92.62	88.59
5	**98.68 ± 0.69**	94.03 ± 2.11	93.38	87.95	98.18	94.98	96.84
6	**99.64 ± 0.36**	96.61 ± 1.86	98.36	97.97	78.78	98.63	99.54
7	75.57 ± 5.67	77.47 ± 1.24	69.52	53.81	**99.38**	64.71	91.94
8	81.29 ± 5.56	56.56 ± 1.53	63.04	54.99	**94.94**	68.78	**81.39**
9	57.35 ± 4.07	58.29 ± 6.58	64.64	38.28	**97.27**	69.33	**90.07**
10	60.00 ± 37.42	**100 ± 0.00**	98.06	98.05	**100.00**	98.77	**100.00**
11	93.93 ± 2.04	80.03 ± 3.93	86.17	84.58	**97.44**	87.78	94.61
12	56.67 ± 8.12	69.55 ± 6.66	69.64	65.80	**100.00**	50.00	90.61
13	—	98.41 ± 0.00	90.70	97.85	**100.00**	**100.00**	**100.00**
14	—	95.00 ± 2.80	17.57	91.30	83.09	48.72	**100.00**
15	—	92.31 ± 0.00	**100.00**	85.71	88.24	72.73	**100.00**
16	—	**100 ± 0.00**	80.00	**100.00**	86.70	80.00	**100.00**
OA(%)	80.19 ± 0.57	69.24 ± 1.56	71.33	65.97	87.92	75.11	**88.51**
AA (%)	72.70 ± 3.76	80.93 ± 1.71	74.83	77.54	**93.79**	78.03	93.50
Kappa	0.7631 ± 0.065	65.27 ± 1.80	67.42	0.6184	0.8625	0.7164	**0.8692**

**Table 8:** classification performance(%) of various GCN methods for Houston University dataset.

Class No.	MiniGCN [31]	DIGCN [31]	DRGCN [32]	GCN [27]	CAD- GCN [16]	MiniGCN [6]	AN-GCN
1	94.85 ± 3.58	93.07 ± 2.73	82.8	88.16 ± 1.90	94.45 ± 3.49	98.39	**100.00**
2	**98.35 ± 1.71**	94.17 ± 2.93	93.38	97.20 ± 0.48	96.43 ± 2.83	92.11	98.34
3	98.09 ± 1.74	95.00 ± 1.68	98.95	97.91 ± 0.13	95.17 ± 4.11	99.6	**100.00**
4	95.60 ± 2.13	90.47 ± 4.09	90.03	96.55 ± 0.41	94.82 ± 2.38	96.78	**99.62**
5	98.64 ± 0.72	100.00 ± 0.00	97.02	89.79 ± 0.71	98.91 ± 1.51	97.73	**100.00**
6	96.58 ± 1.80	94.10 ± 3.86	98.3	98.21 ± 1.15	97.48 ± 3.48	95.1	**100.00**
7	76.05 ± 1.53	**96.06 ± 2.80**	88.77	73.67 ± 1.94	91.58 ± 3.16	57.28	95.94
8	77.28 ± 3.75	73.36 ± 5.63	80.06	65.71 ± 4.64	74.63 ± 4.82	68.09	**97.06**
9	78.98 ± 2.24	**94.33 ± 3.33**	94.18	70.27 ± 3.03	86.75 ± 3.58	53.92	91.76
10	82.92 ± 3.80	88.76 ± 7.63	**99.66**	74.71 ± 2.32	94.24 ± 3.34	77.41	99.23
11	70.07 ± 3.69	90.68 ± 4.32	97.42	75.36 ± 2.37	94.65 ± 2.73	84.91	**97.97**
12	85.87 ± 3.99	87.08 ± 4.25	91.93	79.29 ± 4.80	89.55 ± 1.93	77.23	**97.98**
13	80.93 ± 2.57	92.79 ± 4.34	84.51	12.09 ± 2.68	**96.80 ± 3.68**	50.88	90.88
14	97.73 ± 1.87	**100.00 ± 0.00**	**100**	86.03 ± 3.31	**100 ± 0.00**	98.38	**100.00**
15	99.04 ± 0.76	97.90 ± 1.62	95.07	95.29 ± 1.67	98.02 ± 1.42	98.52	**100.00**
OA(%)	87.00 ± 0.71	91.72 ± 0.64	92.15	80.35 ± 0.61	92.51 ± 0.73	81.71	**97.88**
AA (%)	88.73 ± 0.58	92.52 ± 0.16	92.8	80.02 ± 0.46	93.57 ± 0.60	83.09	**97.92**
Kappa	0.8594 ± 0.077	0.9103 ± 0.069	0.9151	0.7872 ± 0.066	0.9189 ± 0.078	0.8018	**0.9770**

**Table 9:** Number of training and testing samples for the different classes and classification performance (%) of various GCN methods for Botswana dataset.

Class Name	Training	Testing	Class No.	GCN [12]	S^2^GCN [10]	AN-GCN
Water	30	250	1	**100.00**	**100.00**	**100.00**
Hippo grass	30	81	2	98.02	**100.00**	**100.00**
Floodplain grasses 1	30	226	3	98.01	**100.00**	**100.00**
Floodplain grasses 2	30	190	4	97.67	**100.00**	98.38
Reeds	30	244	5	80.67	89.97	**93.72**
Riparian	30	244	6	65.43	92.97	**98.74**
Firescar	30	234	7	96.14	96.60	**100.00**
Island interior	30	178	8	98.03	92.59	**100.00**
Acacia woodlands	30	289	9	80.25	**100.00**	**100.00**
Acacia shrublands	30	223	10	94.76	77.78	**98.17**
Acacia grasslands	30	280	11	86.89	**100.00**	**100.00**
short mopane	30	156	12	86.74	85.11	**100.00**
Mixed mopane	30	243	13	91.42	**100.00**	**100.00**
Exposed soils	30	70	14	82.11	**100.00**	**100.00**
Total	420	2908	AA (%)	89.22	94.45	**99.22**
			OA (%)	89.72	95.36	**99.11**
			Kappa	0.8745	0.9399	**0.9904**

**Table 10:** Classification performance for rice HSI image datasets for different temperature treatments.

Class No	Treatments	168 h	180 h	204 h	216 h	228 h	240 h
1	HDT	0.95	0.99	0.97	1.00	0.98	0.98
2	HDNT1	0.95	0.95	0.98	0.86	0.93	0.97
3	HDNT2	0.81	0.86	0.94	0.94	0.98	0.90
4	HNT	0.95	0.81	0.96	0.89	0.98	0.65
5	Control	0.98	0.99	0.97	1.00	0.95	0.94
	OA	0.93	0.92	0.96	0.93	0.96	0.91
	AA	0.93	0.92	0.96	0.94	0.96	0.89
	Kappa score	0.92	0.90	0.95	0.91	0.95	0.89

**Table 11:** Classification performance for rice HSI datasets for different exposure times.

Class No	Exposure Time (Hours)	HDT	HDNT1	HDNT2	HNT
1	168	0.99	0.74	0.83	0.92
2	180	0.98	0.84	0.90	0.96
3	204	0.98	0.97	0.89	0.89
4	216	0.95	0.71	0.84	0.79
5	228	0.88	0.74	0.88	0.98
6	240	0.96	0.97	0.97	0.95
	OA	0.96	0.81	0.88	0.91
	AA	0.96	0.83	0.88	0.91
	Kappa score	0.96	0.77	0.86	0.89

## Data Availability

https://www.ehu.eus/ccwintco/index.php?title=Hyperspectral_Remote_Sensing_Scenes#Indian_Pines (accessed on 20 October 2022).
